# Optimizing Perfusion-Decellularization Methods of Porcine Livers for Clinical-Scale Whole-Organ Bioengineering

**DOI:** 10.1155/2015/785474

**Published:** 2015-03-31

**Authors:** Qiong Wu, Ji Bao, Yong-jie Zhou, Yu-jia Wang, Zheng-gui Du, Yu-jun Shi, Li Li, Hong Bu

**Affiliations:** ^1^Laboratory of Pathology, West China Hospital, Sichuan University, Chengdu, Sichuan 610041, China; ^2^Department of Pathology and Laboratory of Pathology, West China Hospital, Sichuan University, Chengdu, Sichuan 610041, China; ^3^Laboratory of Pathology and Department of Liver Surgery, West China Hospital, Sichuan University, Chengdu, Sichuan 610041, China

## Abstract

*Aim*. To refine the decellularization protocol of whole porcine liver, which holds great promise for liver tissue engineering. *Methods*. Three decellularization methods for porcine livers (1% sodium dodecyl sulfate (SDS), 1% Triton X-100 + 1% sodium dodecyl sulfate, and 1% sodium deoxycholate + 1% sodium dodecyl sulfate) were studied. The obtained liver scaffolds were processed for histology, residual cellular content analysis, and extracellular matrix (ECM) components evaluation to investigate decellularization efficiency and ECM preservation. Rat primary hepatocytes were seeded into three kinds of scaffold to detect the biocompatibility. *Results*. The whole liver decellularization was successfully achieved following all three kinds of treatment. SDS combined with Triton had a high efficacy of cellular removal and caused minimal disruption of essential ECM components; it was also the most biocompatible procedure for primary hepatocytes. *Conclusion*. We have refined a novel, standardized, time-efficient, and reproducible protocol for the decellularization of whole liver which can be further adapted to liver tissue engineering.

## 1. Introduction

The liver is the largest and most complex glandular organ in the human body. End-stage liver diseases are associated with a high mortality rate [1]. The only effective treatment for hepatic failure is liver transplantation. However, transplantation is limited by severe shortage of donors. Liver tissue engineering is therefore an attractive alternative for organ replacement in patients with liver failure.

Decellularized biological scaffolds provide suitable microenvironments for cell adhesion, survival, and differentiation [2–4]. A decellularized liver could potentially become a tool for stem cell differentiation and maturation in order to eventually engineer autologous liver grafts. These grafts would be bioidentical tissues and organs that could be used for detection of potentially toxic agents in vitro, thereby advancing the progression of safe pharmacologic agents to market; they would also provide viable therapeutic options for individuals awaiting donor organs [5, 6].

Perfusion decellularization allows whole-organ tissue engineering at a clinically relevant scale with an intact organ structure by meeting metabolic demands via intact vasculature and maintaining native ECM-contained cues. Preliminary studies focused on fabrication of implantable tissue-engineered liver using perfusion-decellularized whole-liver scaffolds in rodent models [7, 8]. Generation of perfusion-decellularized native ECM scaffolds, which match human liver in size and structure, would be the first step toward generating functional livers that can be directly transplanted in humans. Whole porcine livers are similar to those in humans with regard to size and anatomy, suggesting that porcine liver could likely be the ideal material source for liver tissue engineering. Decellularization is considered crucial because of the potential to eliminate adverse immune response elicited by cell membrane epitopes, allogeneic or xenogeneic DNA, and damage-associated molecular pattern molecules. Removing cell material, while preserving as much ECM as possible, is essential in the decellularization process. However, all current cell removal agents and methods will alter ECM composition and cause some degree of ultrastructure disruption. Minimization of these undesirable effects is the objective of decellularization. To date, although some studies have been performed on whole-organ porcine liver decellularization [9–11], there is a clear need for the development of more economical and effective protocols.

In our previous studies, we demonstrated that SDS was an essential component for the porcine whole-liver decellularization strategy [12]. To minimize SDS-dependent damage to the ECM, either Triton or SDOC were combined with SDS during the perfusion decellularization process in order to reduce the incubation time. The results showed that SDS combined with Triton achieved an effective and minimally disruptive method for the decellularization of intact porcine whole liver and yielded the best biocompatibility for hepatocyte recellularization. This definition of a standard porcine whole-liver decellularization protocol is a significant step toward a successful regenerative medicine approach to human-scale liver bioengineering for transplantation.

## 2. Materials and Methods

### 2.1. Liver Preparation

All experimental protocols were approved by the Animal Experiment Center of Sichuan University. All animals were cared for in accordance with the requirements of the Laboratory Animal Welfare Act and amendments thereof. Male Bama miniature pigs (Guangxi, China) weighing 10–12.5 kg (*n* = 40) and 25–30 kg (*n* = 6) were obtained from the Animal Experiment Center of Sichuan University (Chengdu, China). Animals were anesthetized with ketamine (6 mg/kg body weight, administered IP, Kelun, Chengdu, China) and xylazine (10 mg/kg IP, Kelun). The abdominal cavity was opened through a long midline incision. The portal vein within the portal triad was skeletonized, dissected to the superior border of the pancreas, ligated proximally, and cannulated. The vena cava was encircled below the level of the liver. A midline sternotomy was performed to expose the suprahepatic vena cava. The liver was then infused with 10 L of perfusate (NaCl 8.3 g/L, KCl 0.5 g/L, HEPES 2.4 g/L, EGTA 0.95 g/L (Sigma, St. Louis, MO, USA)) through the portal vein to remove blood. Both the supra- and infrahepatic vena cava were divided. The liver was excised and infused further on the backbench with perfusate. Attached diaphragmatic muscles and connective tissues were then removed. The hepatic artery and the bile duct were ligated and then the gallbladder was removed. The liver was filled with perfusate and frozen at −80°C until use.

### 2.2. Whole-Liver Decellularization

**Figure 1:**
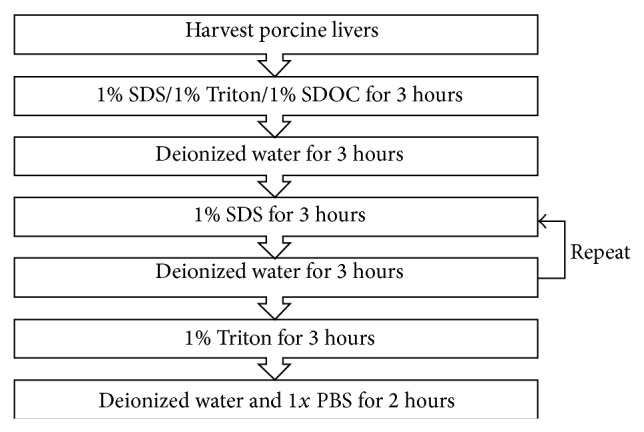
Porcine liver decellularization protocol. SDS: sodium dodecyl sulfate; SDOC: sodium deoxycholate; PBS: phosphate-buffered saline.

On the basis of previous reports, we selected three decellularization detergents, sodium dodecyl sulfate (SDS; Amresco, Solon, OH, USA), Triton X-100 (Triton, Amresco, Solon, OH, USA), and sodium deoxycholate (SDOC; Kelong, Chengdu, China). The frozen livers were thawed at 4°C and placed in a customized glass container. All solutions were perfused through the portal vein. The entire decellularization process was completed at 4°C. The decellularization procedure commenced by infusing 36 L of the 1% detergent in deionized water at a speed of 200 mL/min for 3 hours, followed by infusing deionized water for 3 hours at 200 mL/min. 3 L of 1% detergent was recycled for 3 h at 200 mL/min followed by perfusing deionized water for 3 hours at 200 mL/min. The recycling procedure was then repeated one more time. Subsequently, the liver was washed with 36 L of 1% Triton X-100 in PBS to remove residual SDS, followed by infusion of 36 L of deionized water and 36 L of phosphate-buffered saline (PBS) at 200 mL/min (Figure 1). The content of SDS in the scaffolds was measured by using a colorimetric assay with methylene blue [13].

### 2.3. Histology

Normal fresh liver, decellularized liver matrix, and recellularized liver samples were fixed in 4% paraformaldehyde at room temperature for 24 h. They were dehydrated stepwise using ethanol, immersed in xylene, and embedded in paraffin. The ECM samples were sectioned into 5 *μ*m slides and stained with hematoxylin and eosin (H&E). Sections were mounted in mounting media containing 4′,6-diamidino-2-phenylindole (DAPI) to confirm the extent of decellularization.

### 2.4. Measurement of DNA Content

To measure DNA content, we took decellularized livers and a sample of the fresh frozen liver tissue samples as a control. Briefly, total DNA was isolated from 15 mg of tissue (dry weight) using a commercially available kit (Tiangen Biotech Corporation, Beijing, China). The DNA concentration was estimated at 260 nm using a NanoDrop spectrophotometer (ND-2000c; Thermo, USA) and normalized to the initial dry weight of the samples.

### 2.5. Evaluation of ECM Components

Sections of decellularized liver samples were stained with Masson's trichrome stain, alcian blue stain, elastic fiber stain, and reticular fiber stain following standard protocols. To determine whether collagen I (1 : 1000, rabbit polyclonal IgG, GTX26308; GeneTex, USA), collagen IV (1 : 100, rabbit polyclonal IgG, bs-4595R; Biosson, Beijing, China), laminin (1 : 1000, rabbit polyclonal IgG, GTX11574; GeneTex, USA), and fibronectin (GeneTex, 1 : 100, rabbit polyclonal IgG, GTX72724; GeneTex, USA) were retained in the decellularized matrices, the liver ECM samples were sectioned and stained using immunohistochemistry. Briefly, paraffin sections were rehydrated, incubated in antigen retrieval solution, and stained using antibodies to fibronectin, laminin, and collagen I and collagen IV. Images of the stained slides were captured using an upright microscope (BX51; Olympus, Tokyo, Japan).

### 2.6. Collagen Assay

Collagen was quantified using a colorimetric assay to detect hydroxyproline using a modification of Grant's method [14]. Native liver and the decellularized liver were cut into small pieces, placed in centrifuge tubes, and, subsequently, lyophilized. The samples were weighed, incubated with papain (140 *μ*g/mL) at 60°C overnight, hydrolyzed in 6 M HCl at 115°C for 18 h, and then neutralized, oxidized with chloramine-T, and reacted with* p*-dimethylaminobenzaldehyde. The absorbance at 570 nm was obtained, and a 1 : 10 w/w ratio of hydroxyproline to collagen was used to calculate the collagen content of the tissue. At least 4 parallel samples were analyzed for native and decellularized samples.

### 2.7. Glycosaminoglycans (GAGs) Assay

Sulfated GAGs were quantified using the Blyscan GAG assay kit (B1000; Biocolor, Carrickfergus, UK). The samples were lyophilized, weighed, and then incubated with papain (150 *μ*g/mL) at 65°C for 3 h. The supernatants were placed in 1.5 mL tubes. After Blyscan dye reagent was added, the content of the tubes was homogenized for 30 min and centrifuged for 10 min (10,000 ×g). The deposits were dissolved with dissociation reagent and absorbance was read at 650 nm. At least 4 samples were analyzed for native and decellularized samples.

### 2.8. Hepatocyte Isolation

Primary rat hepatocytes were obtained from male Sprague-Dawley rats (body weight 180–220 g). Hepatocytes were prepared using a two-step collagenase perfusion method, and their viability was confirmed to be >95% by the trypan blue exclusion method. Serum-free medium as described in [15] was used for cell culture. Hepatocytes (0.25 × 10^6^) were plated onto 24-well tissue culture dishes precoated with collagen type I as a hepatocyte culture control (BD Biosciences), according to the methods of Dunn et al. [16]. After cell attachment, monolayers were overlaid with ice-cold medium containing 0.25 mg/mL Matrigel (BD Biosciences) as described elsewhere for collagen-Matrigel sandwich culture [17]. An additional 500 *μ*L of culture medium was supplied and cells were incubated up to 7 days at 37°C and 5% CO_2_. Half of the media volume was changed every day.

### 2.9. Cell Seeding

The decellularized liver scaffold of SDS, T-SDS, and S-SDS groups was sterilized via gamma irradiation. Prior to the perfusion culture experiments, we performed ultraviolet irradiation of whole-organ decellularized grafts for 1 h for decontamination and then transferred them to the sterile cassette, which was connected to the perfusion system through the portal vein and the suprahepatic inferior vena cava. The customized perfusion system consisted of a peristaltic pump, bubble trap, and oxygenator set at 37°C and 5% CO_2_ incubator (Figure S1; see Supplementary Material available online at http://dx.doi.org/10.1155/2015/785474). In order to detect the biocompatibility of scaffold, primary hepatocytes (1 × 10^9^ cells) were injected into the liver grafts through a total of four steps, with 15 min intervals between each step. The cells were allowed to settle and attach to the scaffold for 1 h. Subsequently, the graft was continuously perfused with serum-free medium through the portal vein at a speed of 20 mL/min with continuous oxygenation that delivered an inflow partial oxygen tension of 260 mmHg for 7 days. Half of the media volume was changed every day.

### 2.10. Liver Function Test

Culture medium was collected daily for functional evaluation. Albumin level was tested using ELISA kits (Rat Albumin ELISA Quantitation Set, E110-125, Bethyl) according to the operation manual. Urea concentration was measured using the QuantiChrom urea assay kit (DIUR-500; Bioassay). The absorbance was measured in Sunrise microplate reader (MQX 200; BioTek). The values were normalized to cell number.

### 2.11. Quantitative Real-Time PCR (RT-PCR)

Total RNA was extracted from recellularized liver using TRIzol solution (category number 15596-026, Invitrogen) following the manufacturer's instructions. cDNA of 1 *μ*g total RNA was synthesized using random primers and Prime script reverse transcriptase. Quantitative PCR reactions for the indicated genes were carried out using iScript cDNA Synthesis Kit (category number 170-8890; Bio-Rad) and a fluorescent temperature cycler. Primers sequences were listed in Table 1.

The PCR conditions were 95°C for 30 s, followed by 40 cycles at 95°C for 5 s and 60°C for 5 s (C1000 Thermal Cycler; Bio-Rad). Relative gene expression was quantified according to the comparative Ct method using GAPDH gene as an endogenous control. The melting curve of a product is sequence specific and can be used to distinguish nonspecific from specific PCR products. Gene expression was analyzed with Stratagene analysis software and quantified using the 2^−ΔΔCt^ method.

### 2.12. Statistical Analysis

All data were analyzed using SPSS statistical software (version 17.0). Data were presented as mean ± SEM. One-way analysis of variance (ANOVA) for multiple comparisons was performed to compare datasets. Dunnet-*t* analysis was performed to compare two groups' datasets. A level of *P* < 0.05 was accepted as significant.

## 3. Results

### 3.1. Optimization of the Decellularization Protocol

**Figure 2:**
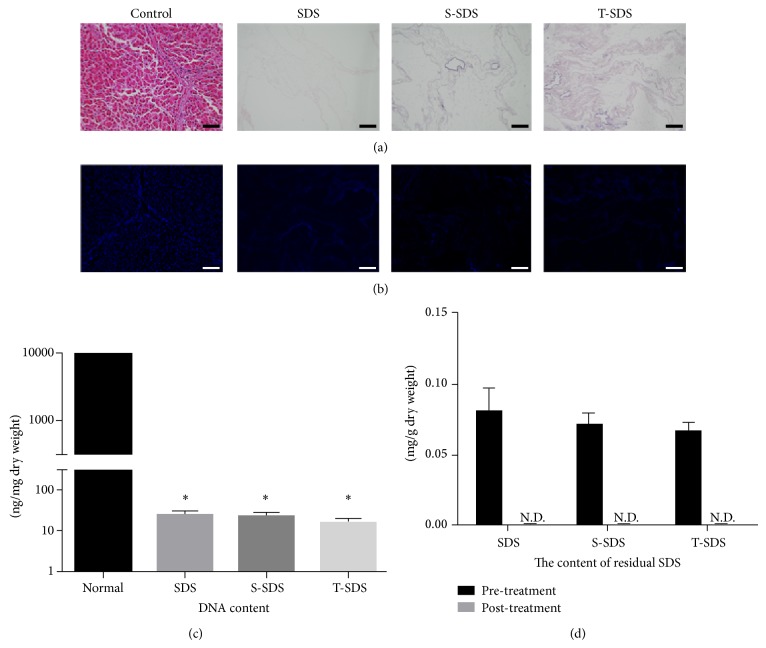
Whole-organ porcine liver decellularization using different detergents. Representative images of (a) hematoxylin and eosin staining and (b) DAPI staining. Scale bars: (a, b) 100 *μ*m. Normal liver was included as a control. (c) The DNA content of the decellularized liver matrix. ^∗^ *P* < 0.05 versus the normal liver group. (d) The content of residual SDS in the decellularized liver matrix of three groups (SDS, S-SDS, and T-SDS) before and after the SDS elimination step via Triton (N.D.: not detected) (*n* = 6 for each group).

Decellularized livers were successfully produced following treatment with either SDS, S-SDS, or T-SDS after portal vein perfusion. After the process of decellularization, all the livers were white and translucent; meanwhile, they maintained their gross appearance and size. Staining with H&E (Figure 2(a)) and DAPI (Figure 2(b)) revealed no visible cell nuclei and cellular material in all groups. The DNA content of normal liver and the dry weight of decellularized livers from the three groups were quantified. The DNA content in normal tissue was 9144.2 ± 97.5 ng/mg, while the DNA content in the SDS group, S-SDS group, and T-SDS group was 25.2 ± 5.3 ng/mg, 23.8 ± 4.7 ng/mg, and 16.8 ± 3.8 ng/mg, respectively, indicating a significant reduction in nuclear material of the whole liver in all groups (*P* < 0.05) (Figure 2(c)). Residual SDS content was nearly negligible in SDS, S-SDS, and T-SDS group liver scaffolds following a 1% Triton wash step (Figure 2(d)).

**Figure 3:**
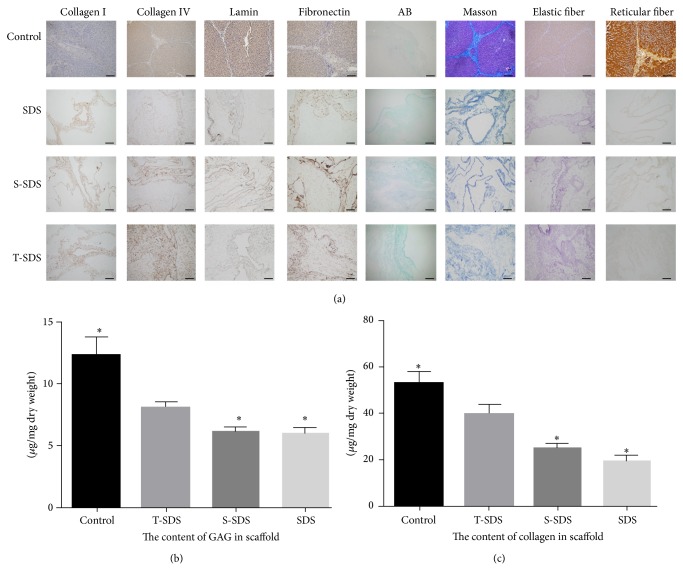
Retention of EMC components by decellularized liver scaffolds after three different SDS-based protocols. (a) Immunohistochemical stain (left 4 panels: collagen I, collagen IV, laminin, and fibronectin) and special stain (right 4 panels: AB staining, Masson's trichrome staining, elastic fiber staining, and reticular fiber staining) of the three types of decellularized liver matrix. Scale bars: 100 *μ*m. (b) The content of GAG in three group scaffolds. (c) The content of collagen in three group scaffolds. Normal liver was included as a control. ^∗^ *P* < 0.05 versus T-SDS group.

Histological and immunohistochemical staining revealed that GAGs, collagen fibers, elastic fibers, reticular fibers, laminin, and fibronectin were preserved in all scaffolds (Figure 3(a)). The amount of remaining collagen and GAGs in the three group scaffolds was also quantified. Scaffolds perfused with T-SDS and S-SDS retained more ECM and GAGs (including total collagen) than those perfused with SDS only. Moreover, the contents of collagens and GAGs were higher in the T-SDS group compared with the SDS-treated scaffolds (100% versus 30% increases). Although there was a significant reduction of GAGs in the decellularized scaffold of T-SDS group, more than 70% of the GAG content was retained, similar to the collagen quantification, which indicates that the combined approach is more appropriate for the fabrication of decellularized liver scaffold (*P* < 0.05) (Figures 3(b) and 3(c)).

### 3.2. Biocompatibility of the Decellularized Liver Scaffolds

**Figure 4:**
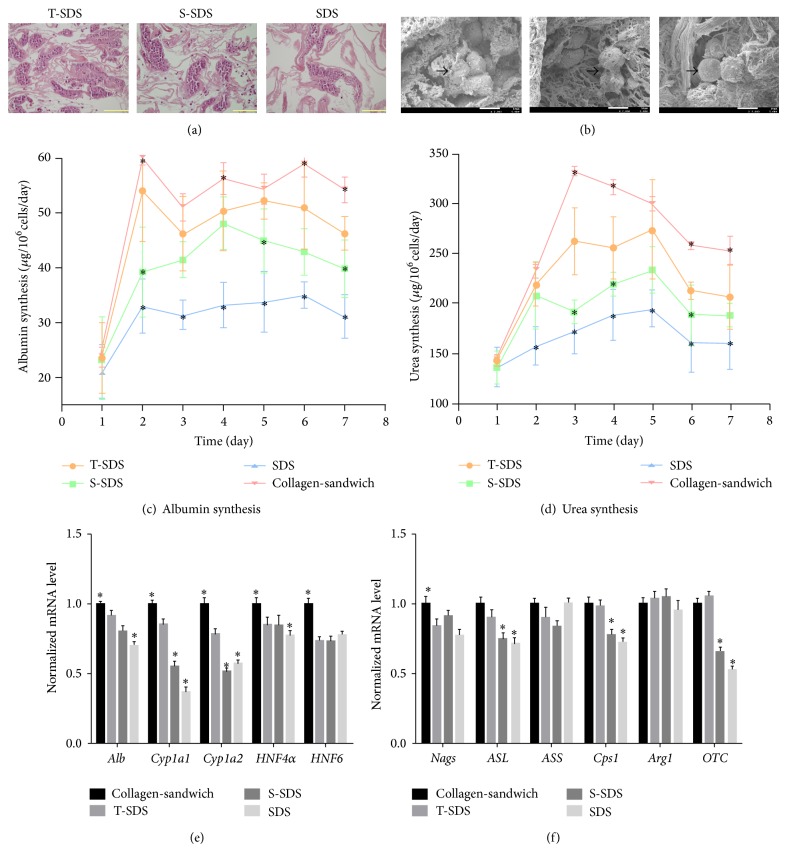
Recellularization of large-scale liver scaffolds prepared by three methods using rat primary hepatocytes after a 7-day perfusion culture. (a) H&E staining, (b) SEM micrographs of recellularized liver graft after 7 days in perfusion culture. (a) Scale bars: 100 *μ*m; (b) arrows indicate hepatocytes, scale bars: 100 *μ*m. (c) Albumin and (d) urea concentration in the culture medium of the collagen-Matrigel sandwich culture and in the perfusion culture medium of three groups of recellularized livers (*n* = 6 each group) during culture. Gene expression of (e) five liver-specific genes (*Alb*,* Cyp1a1*,* Cyp1a2*,* HNF4α*, and* HNF6*) and (f) six genes involved in the urea cycle (*Nags*,* OTC*,* Cps1*,* Ass*,* Asl*, and* Arg1*) were examined by qRT-PCR after 7-day culture. Collagen-sandwich group values served as calibrators to determine the relative expression of each target gene of each group. ^∗^ *P* < 0.05 versus T-SDS group.

In order to assess which type of decellularized liver ECM scaffold is optimal for the initiation and maintenance of liver-specific functions, we manufactured an organ culture device that contains a circulatory system in which the decellularized liver matrix is mounted. Roughly 1 × 10^9^ isolated rat hepatocytes were injected into liver grafts through the portal vein using the perfusion device, while a collagen gel sandwich culture was used as control. Histological staining and SEM revealed that hepatocytes engrafted around the larger vessels and repopulated the surrounding parenchymal area in all groups (Figures 4(a) and 4(b)). Hepatic functions of infused cells were analyzed by quantification of albumin and urea in the medium. Both substances significantly increased when hepatocytes were cultured on T-SDS whole-organ liver ECM as well as on collagen gel sandwich cultures; however, albumin synthesis was relatively low on the S-SDS and SDS scaffolds. The albumin secretion level in collagen gel sandwich, T-SDS scaffold, S-SDS scaffold, and SDS scaffold was 56.3 ± 2.9 *μ*g, 50.4 ± 7.3 *μ*g, 48 ± 4.8 *μ*g, and 33.2 ± 4.1 *μ*g/L × 10^6^ cells/day at day 4, respectively, while the urea synthesis level was 315.9 ± 6.4 *μ*g, 255.1 ± 30.1 *μ*g, 219.3 ± 11.6 *μ*g, and 188.7 ± 25.0 *μ*g/L × 10^6^ cells/day at day 4 (*P* < 0.05) (Figures 4(c) and 4(d)). The expression of five liver-specific genes (*HNF4α*,* HNF6*,* Cyp1a1*,* Cyp1a2*, and* albumin*) as well as the expression of six urea cycle genes (*Nags*,* Cps1*,* Otc*,* Ass*,* Asl*, and* Arg1*) was examined to evaluate hepatocyte functionality of four different groups at day 7. Expression levels were measured by qRT-PCR at day 7. The collagen-sandwich group on day 7 served as the reference point. The T-SDS group had the highest expression levels of three of six urea cycle genes (*P* < 0.05) (*Cps1*,* Otc*, and* Asl*) and three of five liver-specific genes (*P* < 0.05) (*Cyp1a1*,* Cyp1a2*, and* albumin*). The expression of genes in the T-SDS group was similar to the level of the collagen-sandwich groups. The expression of* HNF4α*,* HNF6*,* Nags*,* Ass*, and* Arg1* was similar in all recellularized liver groups (Figures 4(e) and 4(f)). The T-SDS matrix was the most biocompatible and can be used in hepatocyte culture* in vitro*. This suggests that T-SDS-derived intact porcine liver scaffolds have great potential for use in human-scale transplantable liver tissue applications.

### 3.3. Optimization of a Standard Decellularization Protocol for Whole Porcine Liver

**Figure 5:**
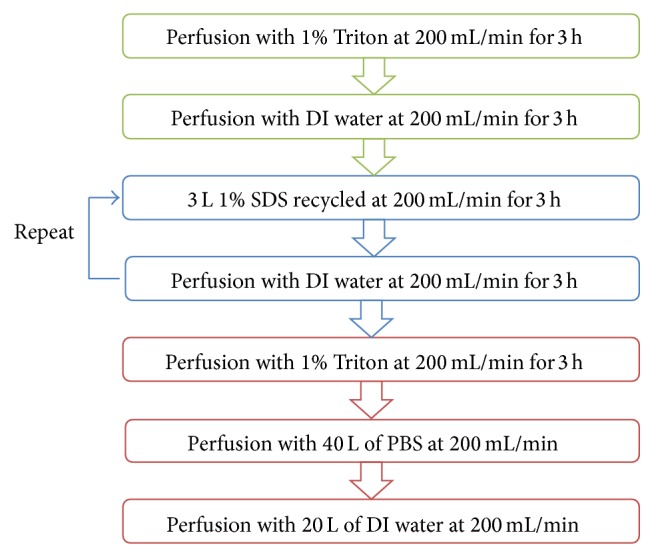
Schematic of standardized decellularization protocol for whole porcine liver. Triton: Triton X-100; DI water: deionized water; SDS: sodium dodecyl sulfate; PBS: phosphate-buffered saline.

After the first freezing-thawing cycle to induce cellular lysis, the standard decellularization protocol was performed as indicated in the flow chart in Figure 5. Whole livers, from pigs that weighed from 10 to 30 kg, were decellularized successfully following this standard protocol (Figure S2).

## 4. Discussion

In the present study, we optimized an effective, minimally disruptive, and standardized protocol for whole-liver decellularization in a large animal model that produces a human-scale three-dimensional liver matrix suitable for supporting functional hepatocytes.

Vascular trees within organs minimize the oxygen diffusion distance to cells. For whole-organ decellularization, perfusion through the vasculature is a remarkably efficient method for the delivery of decellularizing agents to cells and for the transport of cellular material from the tissue. Perfusion decellularization initially generates decellular ECM scaffolds with intact 3D architectures and perfusable vascular networks. This structure can then be recellularized and reendothelialized to regenerate tissues of clinically significant thickness [18]. Whole-organ scaffolds have been generated from cadaveric hearts [19], lungs [20], liver [8], pancreas [21], and kidney [22]. Decellularized rat liver scaffolds, reseeded with primary rat hepatocytes and endothelial cells, produced a metabolically functional whole-liver construct [7, 8, 23].

Freeze-thaw processing which can lyse cells effectively within organs and produce minor disruptions of the ECM ultrastructure is beneficial to decellularization [24]. Perfusion decellularization allows tissue regeneration at a clinically relevant scale with an intact liver structure by meeting metabolic demands via intact vasculature and maintenance of native ECM-contained cues. To date, however, all regenerative efforts based on perfusion-decellularized whole-liver scaffolds have been at the small animal scale [7, 8, 23]. The first step in generating a functional liver that can be directly transplanted in humans will be the generation of perfusion-decellularized native ECM scaffolds that match the human liver in size and structure. Due to the shortage of human donor livers and the ethical and supply restrictions on primate organs, perfusion decellularization of porcine liver is an increasingly attractive option for patient transplants. Although several groups have shown that perfusion decellularization can be applied to porcine liver [9–11], further refinement is required in order to ensure that the scaffolds are of reproducible quality, that they are sterile, and that they are preserved for further processing.

The most effective agents for decellularization of each tissue and organ will depend upon many factors, including the tissue's cellularity, density, lipid content, and thickness [25]. Considering that the liver is the largest internal solid organ and accounts for about 6% of body weight, a balance must be struck during decellularization to stringently remove the cellular component, while at the same time being sufficiently delicate to preserve the ECM. We investigated several commonly used agents, SDS, PAA, Triton X-100, SDOC, and phosphatidase (data not shown), as they have been used successfully to fabricate rodent decellularized livers [26–29]. Only SDS was able to completely decellularize the liver during perfusion [12]. SDS, however, tends to disrupt the native tissue ultrastructure and causes a decrease in the GAG concentration and a loss of collagen integrity [30, 31]. Therefore, we added Triton X-100 or SDOC in order to shorten the SDS perfusion time and thus mitigate tissue damage. Triton X-100 disrupts lipid-lipid and lipid-protein interactions and, to a lesser degree, protein-protein interactions. For tissue delipidation, nonionic detergent Triton is more effective than ionic detergent SDOC [32]. Decellularized liver scaffolds processed from all SDS-based protocols are free of significant DNA content, nuclei, and cytoplasmic proteins. Notably, scaffolds from the Triton + SDS group retained the highest amount of the major ECM proteins (collagen I, collagen IV, laminin, fibronectin, and GAGs). Compared to other studies, we shortened the SDS perfusion time to 6 h, which resulted in less disruption to the ECM and better biologic activity. Above all, the total short procedure time less than 24 h is an obvious advantage compared to previously published reports by others (Table 2).

On the other hand, care must be taken to flush residual SDS from the ECM after decellularization, particularly where it has penetrated into thick or dense tissues. SDS can destabilize the collagen triple helical domain and swell the elastin network; furthermore, some studies have described the cytotoxicity of SDS and focused on methods of residual SDS clearance [33]. Isolated rat primary hepatocytes are generally employed to detect the biocompatibility of liver ECM scaffolds. In our previous studies, we discovered that Triton X-100 was able to eliminate the residual SDS most effectively, as almost no SDS was detected in the ECM [12]. In this study, the SDS-based protocols produced a biological matrix free of any visible cells. However, it was impossible to seed them with rat primary hepatocytes without Triton X-100 washing step, as no cell attachment took place and massive cell lysis occurred within 24 hours of incubation (data not shown). After Triton washing step, this T-SDS-treated scaffold supported primary hepatocyte functions such as synthesis of albumin and production of urea, with gene expression persisting up to 7 days at high level in the perfusion culture system. Cytotoxicity is possible even at reduced SDS concentrations and will inhibit or completely negate the beneficial properties of acellularized ECM scaffold [32, 34]. The residual SDS elimination step is necessary for the whole-liver decellularization protocol to ensure that the produced scaffolds are conducive to recellularization.

Bringing whole-liver scaffolds to clinical quality and scale is only one of many steps toward the regeneration of viable and functional liver. Liver regeneration based on perfusion-decellularized native ECM scaffolds holds great promise for patients suffering from end-stage liver failure, but a series of hurdles must be addressed to allow translation to the bedside. Moreover, the immunogenicity of decellularized porcine liver scaffolds must be carefully assessed in model systems closer to clinical reality. The ideal clinically feasible cell source will be identified through the improvement of our understanding of stem and progenitor cell fate in liver development and diseases.

The data presented here highlight the advantages of the Triton-SDS-Triton strategy for the decellularization of livers of a clinically relevant size. The parameters of initial amount, action time, and residual amount of detergents were significantly reduced compared to previous reports. We have confirmed the utility and reproducibility of our novel strategy in whole-liver decellularization across a range of pig liver sizes. This standardized SDS-based perfusion decellularization protocol can be exploited to develop superior liver scaffolds efficiently, rapidly, and economically, which is ideal for the construction of liver grafts for clinical transplantation. The protocol presented herein can be further adapted to other human-size solid organs.

## Supplementary Material

Figure S1. Representative photographs of the devices for a human-size liver graft (left) and the liver perfusion culture system in an incubator (right).
Figure S2. Decellularized whole liver scaffold from a 30kg pig following a standardized human-scale whole-liver decellularization protocol. The macrographs of liver (A) before and (B) after decellularization. Scale bars: 10 cm. (C) H&E staining of decellularized liver . Scale bar: 100 μm.

## Figures and Tables

**Table 1:** RT-PCR primer sequences.

Gene	Forward primers	Reverse primers
Alb	GGCACCAAGTGTTGTACCCT	AGCACACACAGACGGTTCAG
Cyp1a1	AGCTAATCAAAGAGCACTACAGG	CCTTATCATCTGAGAGCTGG
Cyp1a2	GAGAAGGTGATGCTCTTCGG	ATGCAGGAGGATGGCTAAGA
HNF4*α*	CCTTGGACCCAGCCTACA	GCTTGAGGCTCCGTAGTGT
HNF6	CCTGGAGCAAACTCAAGTCC	CCGTGTTCTTGCTCTTTCC
Otc	TGAGGATCCTGCTCAACAAG	ACGGCCTTTCAGCTGTACTT
NAGS	CCGTTCGGTGCTTCTAGACT	CAGGTTCACATTGCTCAGGA
Arg1	CAACACTCCGCTGACAACC	CAGATATGCAGGGGGTCAC
Asl	TCAACAGTATGGATGCCACC	CAAAGTTGAATTCCTTGGTACC
Ass	CCAGGAAGAAGGCACTGAAG	GCCTAGGAGATAGCGGTCCT
Cps1	ACATTGGCTGCAGAATACCC	ACAGCCCAGCACCATTATTC

**Table 2:** Procedure of whole porcine liver decellularization of other investigators.

SDS concentration	Action time	References
0.25% + 0.5%	48 h + 3 h	[9]
0.01% + 0.1% + 1%	24 h + 24 h + 48 h or more	[10]
1%	16 h	[11]
