# Supplementary material for: Optimizing Perfusion-Decellularization Methods of Porcine Livers for Clinical-Scale Whole-Organ Bioengineering
Source: Biomed Res Int. 2015 Mar 31;2015:785474. doi: 10.1155/2015/785474 (PMC4396818; doi:10.1155/2015/785474)
Supplement: Supplementary file 1 — Figure S1. Representative photographs of the devices for a human-size liver graft (left) and the liver perfusion culture system in an incubator (right). Figure S2. Decellularized whole liver scaffold from a 30kg pig following a standardized human-scale whole-liver decellularization protocol. The macrographs of liver (A) before and (B) after decellularization. Scale bars: 10 cm. (C) H&E staining of decellularized liver . Scale bar: 100 μm. [file 785474.f1.pdf]

Supplementary Data

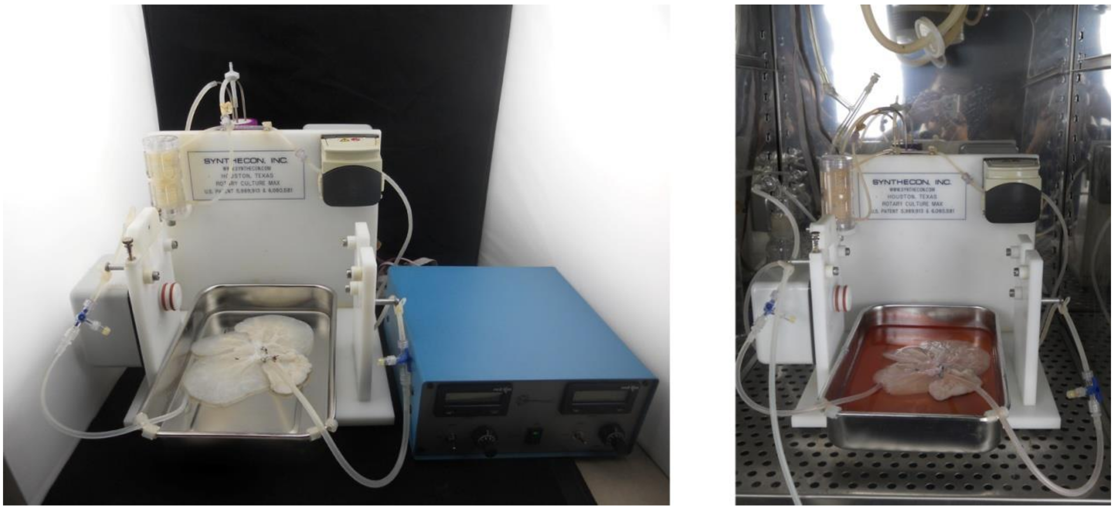

Figure S1. Representative photographs of the devices for a human-size liver graft (left) and the liver perfusion culture system in an incubator (right).

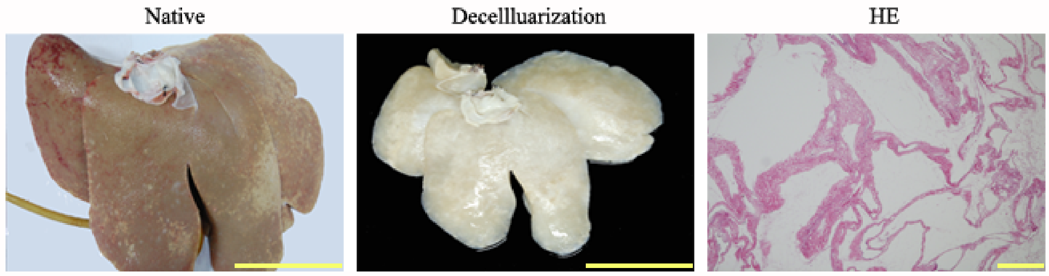

Figure S2. Decellularized whole liver scaffold from a 30kg pig following a standardized human-scale whole-liver decellularization protocol. The macrographs of liver (A) before and (B) after decellularization. Scale bars: 10 cm. (C) H&E staining of decellularized liver. Scale bar: 100 µm.
